# Water intake pancreatic ultrasonography improves pancreatic body and tail visualization: contributing factors revealed by quantitative analysis

**DOI:** 10.1007/s10396-026-01622-y

**Published:** 2026-05-09

**Authors:** Akihiko Okamura, Toshiaki Takahashi, Masaki Nemoto, Yuki T. Okamura

**Affiliations:** 1 Sainooka Clinic, 416–5 Jimbohara, Kamisato-machi, Kodama-gun, Saitama, Japan; 2 Takahashi Naika Clinic, 9436–1 Irino-cho, Chuo-ku, Hamamatsu-shi, Shizuoka, Japan; 3 Nemoto Naika Clinic, 1123 Tenryugawa-cho, Chuo-ku, Hamamatsu-shi, Shizuoka, Japan

**Keywords:** Pancreatic ultrasonography, Fluid-filled stomach, Water intake pancreatic ultrasonography (WPUS), Body mass index, Pancreatic Image Scale (PIS)

## Abstract

**Purpose:**

Transabdominal pancreatic ultrasound is simple and noninvasive; however, the image quality is often degraded by visceral fat and gastrointestinal gas. Water intake pancreatic ultrasonography (WPUS) may help address this limitation, but quantitative evidence remains limited. We evaluated the effects of WPUS and examined the 10-grade Pancreatic Image Scale (PIS) as a quality control metric.

**Methods:**

We retrospectively reviewed the medical records of 581 patients who underwent WPUS. The PIS (0–9) was recorded before and after water intake, and improvements were analyzed for associations with body mass index (BMI), age, and sex to identify factors influencing pancreatic visualization. We added a reproducibility study in which three gastroenterologists independently re-scored 30 anonymized cases three times.

**Results:**

WPUS improved pancreatic visualization in 82% of cases; the proportion achieving PIS ≥ 6 increased from 17% in pre-water supine scans to 54–71% after WPUS (*P* < 0.001). Higher BMI was associated with lower baseline PIS (*β* =  − 0.12, *P* < 0.001). WPUS-related improvement was significantly greater in women than in men (*β* = 0.99, 95% CI 0.56–1.42, *P* < 0.001). PIS demonstrated moderate-to-good repeatability (intra-reader ICC 0.80; inter-reader ICC 0.71).

**Conclusion:**

Factor analysis indicated no sex difference in pre-WPUS pancreatic visualization, but women exhibited greater improvement after water intake. WPUS significantly improved visualization of the pancreatic body and tail, as reproducibly quantified using PIS. These findings support WPUS as a practical tool for routine clinical assessment and as a potential adjunct to early detection strategies for pancreatic disease.

**Supplementary Information:**

The online version contains supplementary material available at https://doi.org/10.1007/s10396-026-01622-y.

## Introduction

Pancreatic cancer is often diagnosed at an advanced stage, making early detection challenging. Transabdominal ultrasonography (US) is less sensitive than computed tomography (CT) or magnetic resonance imaging (MRI), yet its noninvasiveness, bedside availability, and low cost support its widespread use in routine screening. However, image quality is highly dependent on body habitus, and gastrointestinal gas and visceral fat particularly impair visualization of the pancreatic body and tail [1–3]. Several approaches have been developed to optimize pancreatic US imaging. Postural changes can reposition the pancreas and dislodge the gases and contents in digestive organs from the region of interest [4, 5]. Another practical method is the fluid-filled stomach technique, commonly referred to as water intake pancreatic ultrasonography (WPUS), in which ingested water displaces gastric gas and creates an acoustic window. At our institution, water intake is routinely combined with positional changes to maximize visualization of the pancreatic body and tail. Several studies have shown improved visibility with WPUS, but most were restricted to high-risk or small cohorts and relied on qualitative assessment [6–11]. This study aimed to (1) quantify, in a large, unselected outpatient cohort, the depictable range using the Pancreatic Image Scale (PIS) and identify clinical determinants of WPUS-related improvements (BMI, age, sex); and (2) examine the intra- and interobserver reproducibility of PIS in a separate, smaller reader study.

## Materials and methods

### Study design

This retrospective, cross-sectional study was divided into two components. Study 1 analyzed routine WPUS examinations annotated with the newly developed PIS. Study 2 assessed the intra- and interobserver reproducibility of the PIS in an independent reading experiment involving three experienced gastroenterologists. Figure 1 shows the study flow diagram and patient selection process for both studies, with additional details on case selection and exclusion provided in Supplementary Figure S1. The primary evaluation metric was the PIS, a 10-point ordinal scale that assesses the depictable range of the pancreas (Fig. 2). Statistical analyses were performed to assess the factors associated with PIS values and their improvement.

**Fig. 1:**
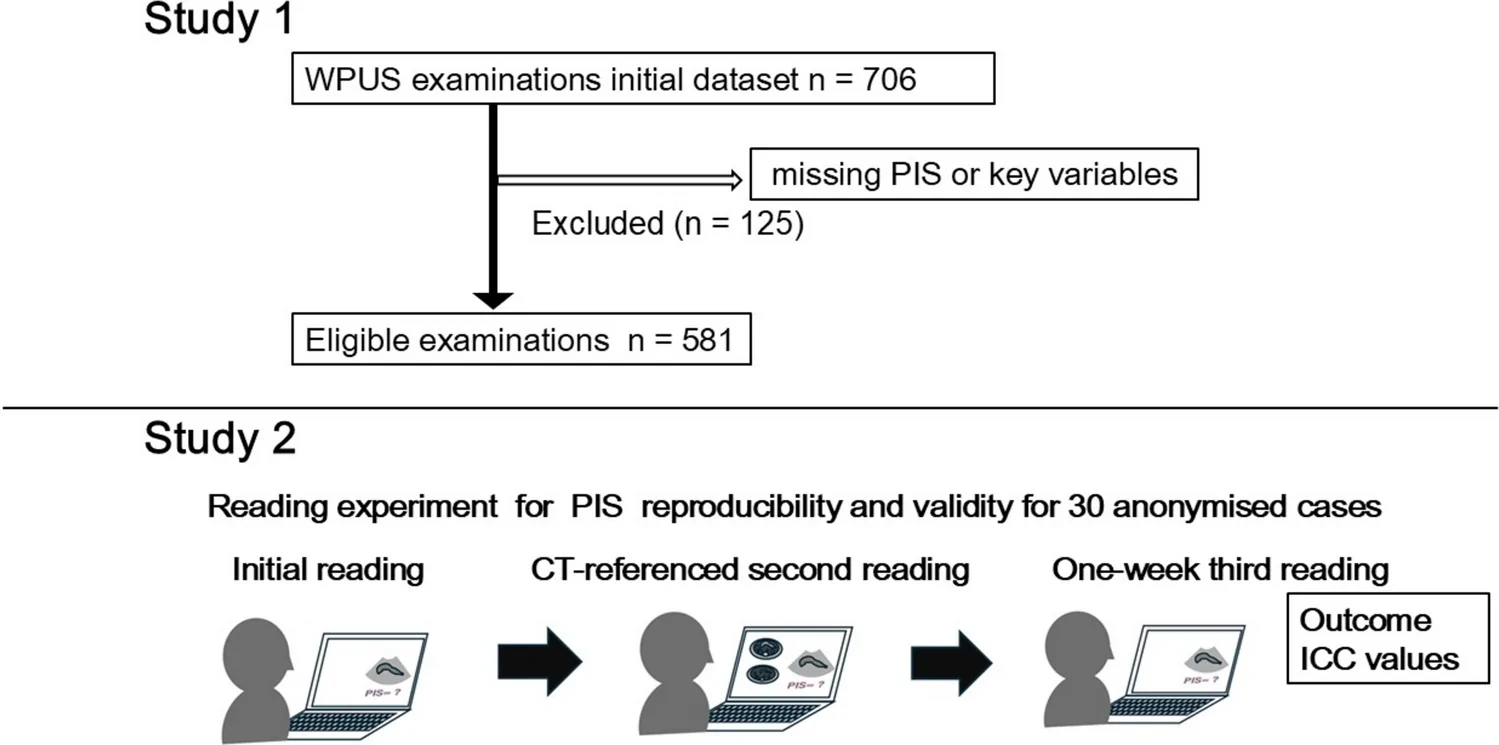
Study flow diagram. This study consisted of two complementary parts. Study 1 was a retrospective analysis of clinical records, and Study 2 was a controlled reading experiment designed to confirm the reproducibility of the Pancreatic Image Scale. Study 1: A total of 706 consecutive water intake pancreatic ultrasonography examinations were retrospectively identified. After excluding 125 records with missing PIS data or other critical variables, 581 examinations were included in the primary analysis. A detailed version of the study flow is available in Supplementary Fig. S1. Study 2: A reading experiment assessed the reproducibility and validity of the PIS, comprising three steps: an initial reading, a CT-referenced second reading, and a third reading performed one week later. The central illustration shows computed tomography images used for re-evaluation. Abbreviations: PIS, Pancreatic Image Scale; WPUS, water intake pancreatic ultra sonography; CT, computed tomography; ICC, intraclass correlation coefficient

**Fig. 2:**
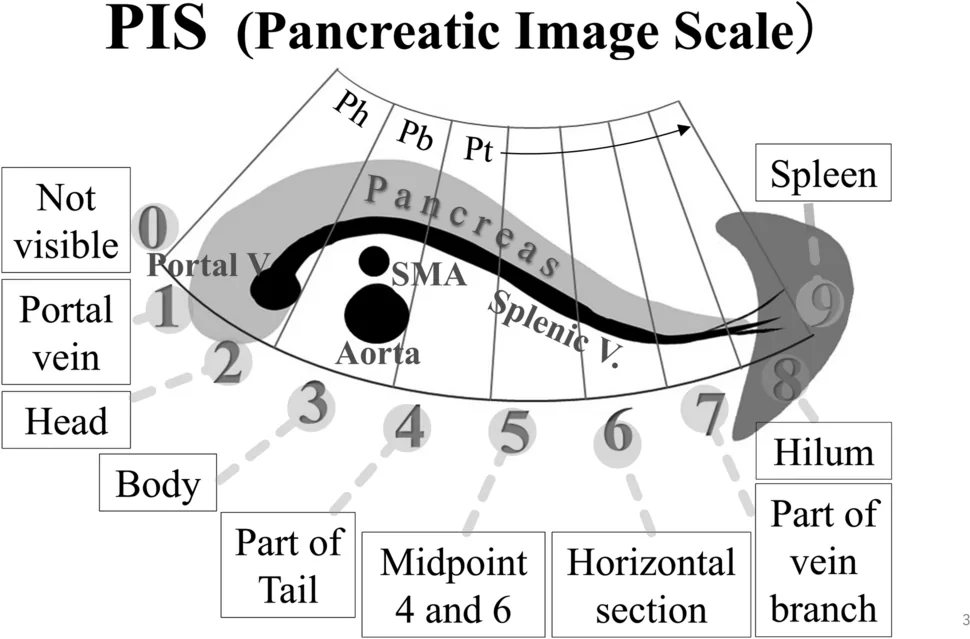
Pancreatic Image Scale (PIS). The PIS is a 10-level ordinal scale (0–9) used to evaluate the extent of pancreatic visualization during ultrasonography. A score of 0 indicates no visualization of the pancreas, while a score of 9 represents complete visualization, including the splenic hilum. The scale progresses from relatively easy-to-observe areas, such as the pancreatic head and body, to more challenging, deeper regions, reflecting the increasing difficulty of visualization. Lower PIS scores represent progressively reduced visualization of the pancreatic tail. Abbreviations: SMA, superior mesenteric artery; PIS, Pancreatic Image Scale; Ph, pancreatic head; Pb, pancreatic body; Pt, pancreatic tail

### Participants

Between January 2012 and December 2022, 706 consecutive WPUS examinations were retrospectively identified at our outpatient clinic. Patients who had undergone total gastrectomy were excluded, whereas three patients with partial gastrectomy were included. WPUS was intended for all outpatients undergoing abdominal ultrasound (AUS) during routine care, regardless of surgical history or postoperative anatomical alterations. In practice, WPUS was performed for all examinations unless clinically inappropriate (e.g., aspiration risk, nausea, or inability to drink water). Although case identification required manual review of paper charts, all US images were digitally archived and available for re-evaluation. After excluding 125 records with missing PIS or other critical variables, 581 examinations were included in Study 1 (258 men and 323 women; baseline characteristics are summarized in Table 1).

**Table 1:** Baseline characteristics of the study cohort (*n* = 581)

Characteristic	Men	Women	*P*‐value
*N*, *n* (%)	258 (44.4%)	323 (55.6%)	–
Age, years, median [IQR]	58 [46–69]	64 [50–72]	0.055
Height, cm	168.1 (6.3)	154.9 (8.0)	< 0.001
Body weight, kg	68.4 (13.1)	55.8 (12.3)	< 0.001
BMI, kg/m^2^	24.2 (4.4)	23.2 (4.4)	0.043

Continuous variables are presented as mean (SD) or median [IQR], and categorical variables as *n* (%). Percentages are based on non-missing denominators. *P* values represent simple univariate comparisons between men and women and are provided solely for descriptive purposes

Abbreviations: BMI, body mass index; SD, standard deviation; IQR, interquartile range

During the initial study period, WPUS was performed in seven body positions—pre-WPUS: sitting (Sit), semi-Fowler (SF), and supine (SP); and post-WPUS: Sit, SF, SP, and right-lateral decubitus (RLD). To shorten examination time, the baseline assessment was later limited to the SP position only, yielding a five-position protocol (pre-WPUS SP; post-WPUS Sit, SF, SP, and RLD). Both protocol cohorts were retained. All 581 examinations were analyzed for overall pre- and post-WPUS comparisons (Fig. 3). Of the seven-position cases, 263 examinations had complete positional analysis data (Fig. 4).

**Fig. 3:**
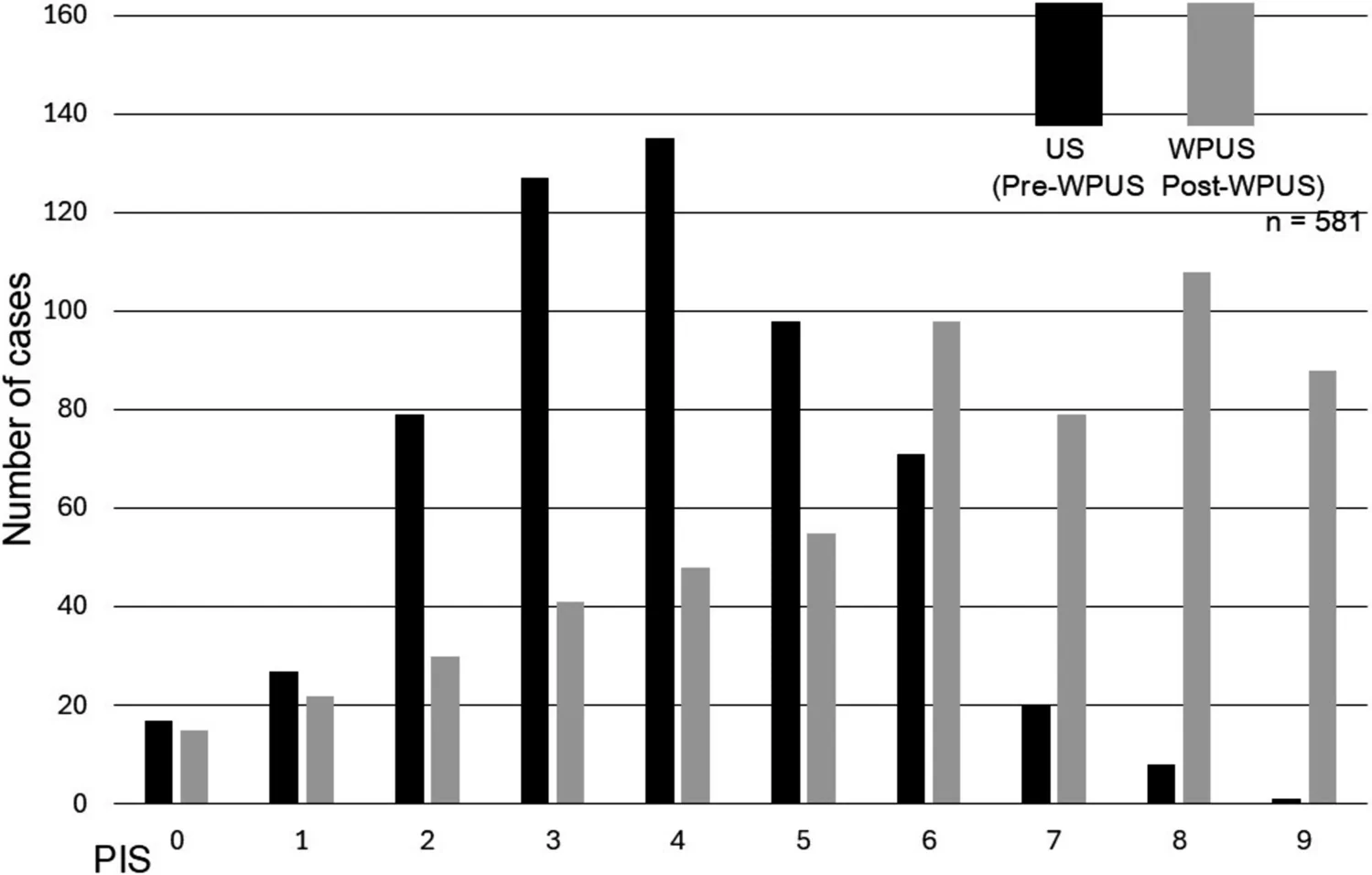
Frequency distribution of PIS before and after WPUS. The figure shows the frequency distribution of PIS before WPUS (SP, black bars) and after WPUS (WSP, grey bars). PIS values increased after water intake, indicating improved pancreatic visualization (*n* = 581). Abbreviations: PIS, Pancreatic Image Scale; WPUS, water intake pancreatic ultrasonography; SP, score before WPUS; WSP, score after WPUS

**Fig. 4:**
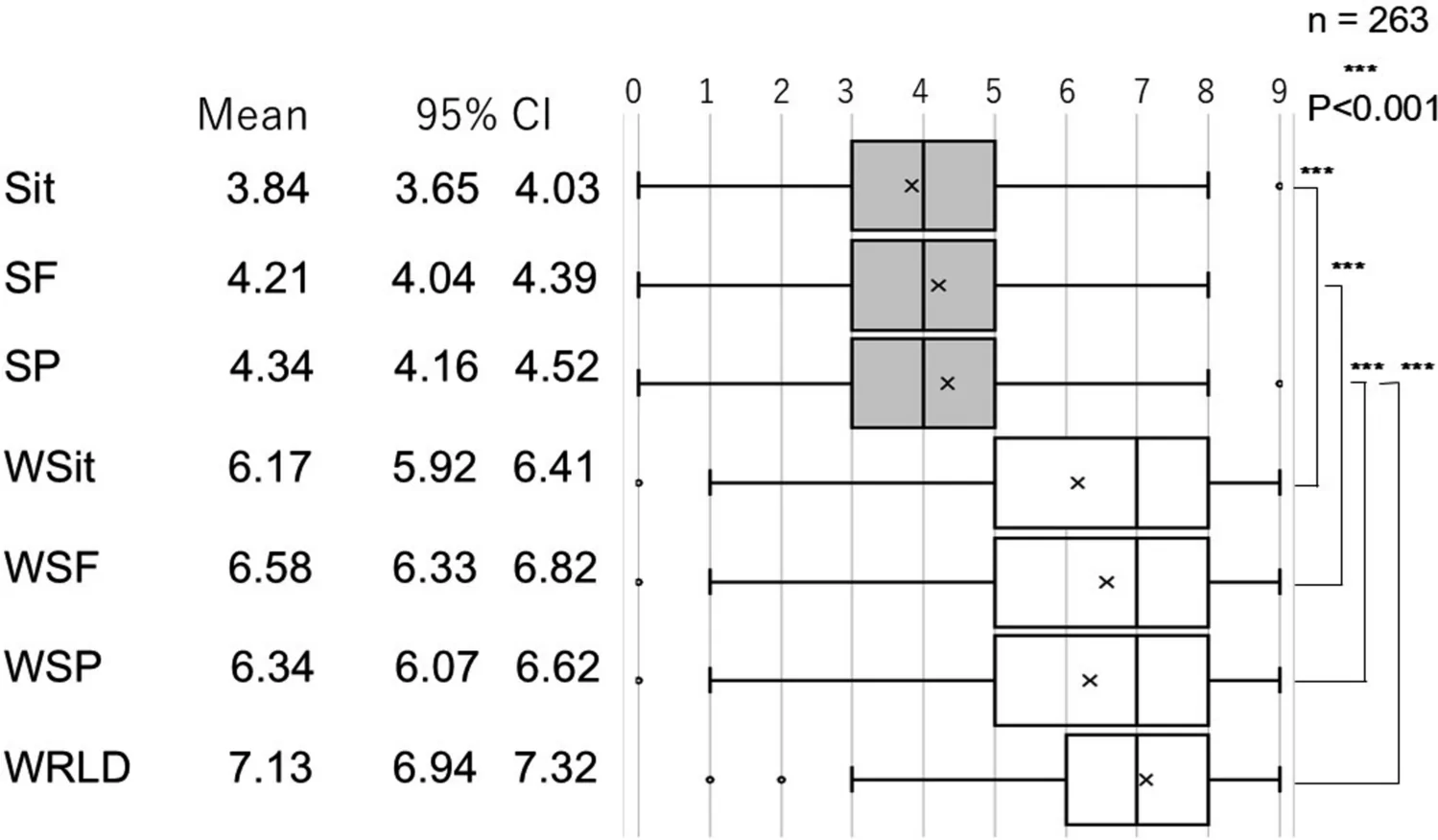
Box-and-whisker plots of PIS by body position. Pre-water positions: Sit, sitting; SF, semi-Fowler; SP, supine. Post-water positions during water intake pancreatic ultrasonography: WSit, sitting; WSF, semi-Fowler; WSP, supine; RLD, right-lateral decubitus. The boxes show the interquartile range (IQR) from the 25th percentile (Q1) to the 75th percentile (Q3), with a horizontal line indicating the median. Whiskers extend to values within 1.5 × IQR, and outliers are shown as points. The “×” marks represent the mean for each group. ***P < 0.001 (Wilcoxon signed-rank test); *n* = 263. Abbreviations: PIS, Pancreatic Image Scale; WPUS, water intake pancreatic ultrasonography; Sit, sitting; SF, semi-Fowler; SP, supine; WSit, sitting after water intake; WSF, semi-Fowler after water intake; WSP, supine after water intake; RLD, right-lateral decubitus; IQR, interquartile range

For correlation analysis between BMI and PIS improvement, the baseline PIS required averaging Sit, SF, and SP positions, which was available only in the seven-position protocol. Therefore, 90 examinations that included both seven-position PIS and BMI data were analyzed. In contrast, the multivariable analysis evaluating predictors of WPUS improvement used the SP PIS as the pre-WPUS baseline, allowing inclusion of 249 examinations from both seven- and five-position protocols. For patients with multiple WPUS examinations, datasets were harmonized as follows: if a BMI measurement was available, the scan closest in time to that BMI measurement was retained; otherwise, the most recent examination was selected for the primary analysis.

For the descriptive summary of lesion detection (Table 2), the full set of 706 consecutive WPUS examinations was used, because lesion ascertainment did not require complete covariate data such as PIS or BMI.

**Table 2:** Pancreatic lesions identified in this study and their confirmation modality (*n* = 14)

No.	Location	Size, mm	Echogenicity	First detected	Diagnosis	Verification modality	Comments
1	Head	8	Cystic	US	IPMN-BD suspected	MRCP	–
2	Head	45	Solid	US	Malignancy-suspected lesion associated with IPMN (no pathology)	MRCP/CT	Not surgically resected
3	Head	15	Cystic	US	IPMN-BD suspected	MRCP	–
4	Head (uncinate)	20	Cystic	US	IPMN-BD suspected	MRCP	Multilocu-lated
5	Head (uncinate)	12	Cystic	WPUS	IPMN-BD suspected	MRCP	Detected by shifted WPUS windows
6	Body	12	Cystic	WPUS	IPMN-BD suspected	MRCP	–
7	Body	25	Solid	US	Pancreatic neuroendocrine tumor	MRCP/CECT	Surgically resected; Pathology confirmed
8	Body	7	Cystic	WPUS	IPMN-BD suspected	MRCP	–
9	Body	5	Cystic	US	IPMN-BD suspected	MRCP	With additional lesions
10	Body	8	Cystic	US	Cyst or IPMN suspected	MRCP	Multiple lesions; additional 6-mm cyst in the head (uncinate)
11	Body	10	Cystic	US	IPMN-BD suspected	MRCP/CECT	–
12	Body	8	Cystic	WPUS	IPMN-BD suspected	MRCP	–
13	Tail	8	Cystic	WPUS	Not confirmed	None	–
14	Tail	9	Cystic	WPUS	IPMN-BD suspected	MRCP	–

“First detected” indicates the imaging modality at the same visit in which the lesion was first visualized. Lesion verification followed routine clinical practice rather than a unified protocol. One WPUS-detected lesion (No. 13) lacked confirmatory imaging because follow-up information was unavailable. All cystic lesions in this table were clinically suspected and not pathologically confirmed

Abbreviations: US, conventional transabdominal ultrasound; WPUS, water intake pancreatic ultrasonography; CT, computed tomography; MRCP, magnetic resonance cholangiopancreatography; CECT, contrast-enhanced computed tomography; IPMN-BD, intraductal papillary mucinous neoplasm, branch-duct type

### Ethics approval and consent

This retrospective study was conducted in accordance with the Declaration of Helsinki 1964 (and later revisions), and was approved by the Ethics Committee of the Hamamatsu University School of Medicine (Approval No. 21000114). Given its observational design using existing clinical records, the requirement for written informed consent was waived. Instead, study details were disclosed to patients via an on-site information sheet and the clinic website, with an opt-out opportunity before data analysis. This study is reported in accordance with the Strengthening the Reporting of Observational Studies in Epidemiology (STROBE) guidelines.

### PIS definition

The PIS (as shown in Fig. 2) is an ordinal 0–9 scale for quantifying the anatomical extent of pancreatic visualization. In this scale, each PIS value is referred to as a score. Score 0 indicates complete non-visualization. Score 1 corresponds to visualization of the pancreatic head based on identification of the portal vein. For subsequent scores, intermediate values increase proportionally to the anatomical extent visualized. Score 3 is defined as visualization of the pancreatic body, recognized between the left margin of the superior mesenteric–portal vein confluence and the left margin of the aorta [12, 13]. Score 4 represents visualization of the initial portion of the pancreatic tail, located at the “shoulder” just superior to the left lateral contour of the aorta and appearing as a straight segment with uniform parenchymal thickness. Beyond this region, vascular landmarks become less prominent; therefore, tracing the pancreatic parenchyma along the splenic vein, aided by the gradual tapering and gentle S-shaped curvature of the distal tail, is used to identify further extension of the tail. Score 6 is defined as the visualization of the horizontal segment of the splenic vein. Score 7 corresponds to the level at which a part of a splenic vein branch becomes identifiable. Score 9 indicates complete and confident delineation of the pancreatic tail at the splenic hilum. Scores not explicitly defined were determined as the midpoint between adjacent landmark levels.

### Imaging procedure

All examinations were performed by a board-certified gastroenterologist with 25 years of experience (AO) using HI VISION Avius (Hitachi, Tokyo, Japan) or ARIETTA 750 (FUJIFILM, Tokyo, Japan) scanners with convex probes (2–5 MHz). The sensitivity time control and time gain compensation (STC/TGC) ratio was manually adjusted to obtain uniform gain within an imaging depth of 6–18 cm. Other imaging parameters—including focal zone, frequency selection, and dynamic range—were kept in the default automatic mode recommended by each US system manufacturer. All examinations were performed under the same settings throughout the study period. US techniques other than WPUS followed the Japan Society of Ultrasonics in Medicine Manual for Abdominal Ultrasound in Cancer Screening and Health Checkups (first edition, 2011; current edition, 2021) [4].

### WPUS protocol

Before water intake, all patients underwent standard AUS, which served as the “baseline US” for comparison with WPUS. After baseline US, patients ingested approximately 500 mL of boiled and degassed barley tea (or water when tea was contraindicated), cooled to near body temperature. Boiling once to remove microbubbles was performed to improve the acoustic window. The liquid was consumed over 4–6 min, while the operator monitored tolerance. After ingestion, scanning was repeated in the transverse epigastric and left subcostal approaches. An experienced operator supervised all examinations to ensure safety and maintain consistent timing. For documentation, two representative still images were saved for each body position; however, the examiner assigned a single PIS score per position based on the overall live-scan assessment. Patient posture was adjusted using an electrically tilting examination table. Examination timeline: (1) Baseline pancreatic visualization was performed in the sitting, semi-Fowler, and supine positions. (2) The patient then ingested the prepared liquid as above. (3) After completion, post-WPUS scanning repeated the same positions in the same order, followed by right lateral decubitus (RLD) to utilize the water-filled stomach, which shifts to the right lateral side, providing a different scanning approach.

### Statistical analysis

The primary analysis focused on the change in PIS from pre- to post-WPUS across different body positions. Secondary outcomes included associations between PIS values (and PIS improvement, defined as the difference between pre- and post-WPUS scores) and patient characteristics (BMI, age, and sex). Changes in the PIS were evaluated using the Wilcoxon signed-rank test. Associations between PIS (or PIS improvement) and patient characteristics were assessed using Spearman’s rank correlation coefficient. Given the large sample size, assumptions of linearity and homoscedasticity were considered sufficiently robust and were assessed visually based on score distributions. Both PIS and PIS improvement were treated as continuous variables and analyzed using multivariable linear regression to investigate the independent effects of patient characteristics. Robustness was further tested using binary logistic regression to examine sex differences in PIS improvement. A two-sided *P* < 0.05 was considered statistically significant. All analyses were conducted using EZR v1.65 (graphical interface for R 4.2.0) [14].

### Inter-observer reliability (Study 2)

Three gastroenterologists (clinical experience of 6, 15, and 22 years) participated as blinded readers. All readers routinely perform abdominal US in daily practice. After a brief consensus-based training session using 20 reference static images with predefined PIS labels, each reader independently scored 30 additional examinations in three randomized reading phases (baseline, CT-referenced, and repeated reading after 1 week). Only representative static US frames were used to ensure identical visual information for all readers. Cine loops were intentionally not used. For the CT-referenced phase, axial CT images were shown side-by-side when CT data were available; randomization of cases was performed within this CT-available subset. Readers were blinded to clinical information and to each other’s ratings, and case order was re-randomized for each phase. Intra- and inter-observer reproducibility were assessed using intraclass correlation coefficients (ICC (1,1) and ICC (3,1)) [15]. ICC values were interpreted as poor (< 0.50), moderate (0.50–0.75), good (0.75–0.90), and excellent (> 0.90), according to Koo and Li [16].

## Results

### Study 1

Patient characteristics of the study cohort are summarized in Table 1. The cohort consisted of 581 examinations (Study 1). Figure 3 compares the PIS frequency distributions obtained in the SP position before and after water intake (*n* = 581), showing a distinct rightward shift following WPUS. In the seven-position subset (*n* = 263), 17.1% of cases achieved a PIS ≥ 6 at pre-WPUS. In contrast, at post-WPUS, this proportion rose to 53.7% in the Sit position, 62.2% in SF, 63.7% in SP, and 71.0% in RLD. Overall, 82.7% of examinations reached a PIS ≥ 6 in at least one post-WPUS position. Figure 4 shows mean PIS values with 95% confidence intervals (CIs) and box-and-whisker plots for each position. Significant increases were observed at every position post-WPUS (Wilcoxon signed-rank test, *P* < 0.001). The RLD position also yielded significantly higher scores than the pre-WPUS SP. These results quantitatively support the consistent enhancement of pancreatic visualization achieved with WPUS.

Figure 5 presents scatterplots of BMI and PIS improvement. BMI was significantly negatively correlated with PIS before and after WPUS (Spearman’s *ρ* =  − 0.40 and − 0.41, respectively; *P* < 0.001). The correlation with PIS improvement was weak and not significant (*ρ* =  − 0.19, *P* = 0.07). Given the large sample size, assumptions of the linear regression, including linearity and homoscedasticity, were visually assessed using score distributions (Figs. 3 and 5). These inspections indicated approximate normality and homoscedasticity, supporting the treatment of PIS as a continuous variable and justifying the use of multivariable linear regression in subsequent analyses.

**Fig. 5:**
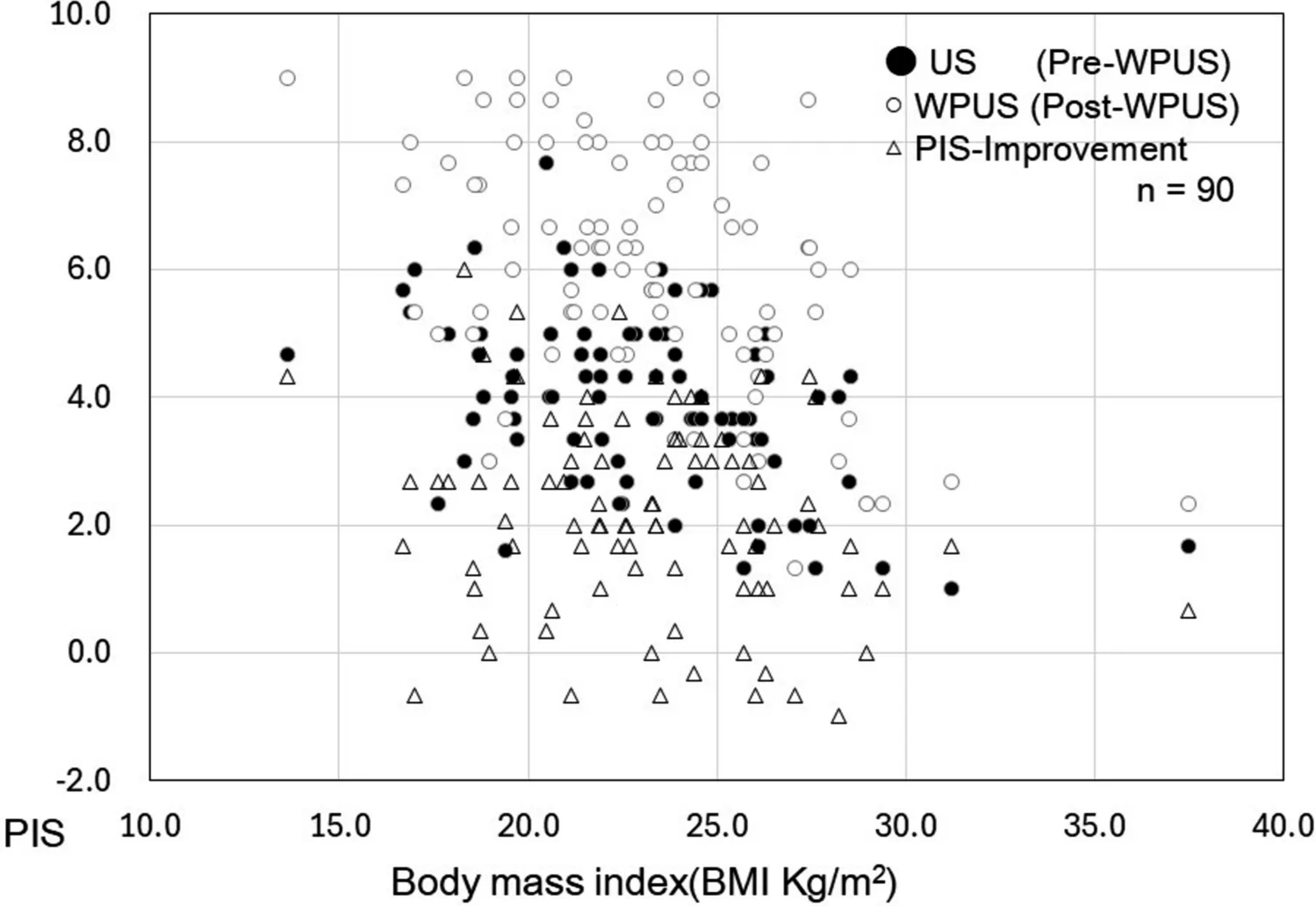
Scatter plots of BMI and PIS before and after WPUS, and improvement with WPUS. BMI versus PIS at pre-WPUS (●), post-WPUS (○), and PIS improvement (post-WPUS − pre-WPUS; △). Significant negative correlations were observed between BMI and PIS in both pre- and post-WPUS (Spearman’s *ρ* =  − 0.40 and − 0.41, respectively; *P* < 0.001). The correlation with PIS improvement was weak and not significant (*ρ* =  − 0.19, *P* = 0.07; *n* = 90). Abbreviations: BMI, body mass index; PIS, Pancreatic Image Scale; WPUS, water intake pancreatic ultrasonography

In the pre-WPUS examination, female patients showed higher PIS values than male patients (Supplementary Fig. S2). This difference was explained by the lower BMI distribution among female patients (Supplementary Fig. S3). We conducted a multivariable linear regression analysis assuming PIS to be a continuous variable with equal intervals. Table 3 summarizes the effects of BMI, sex, and age on pre-WPUS PIS and on PIS improvement. Regarding pre-WPUS SP PIS values, age (*β* =  − 0.02, *P* < 0.001) and BMI (*β* =  − 0.12, *P* < 0.001) were significant negative predictors, whereas sex showed no significant association (*β* = 0.3, *P* = 0.14). Although the influence of age was statistically significant, the effect size was small. These findings indicate that BMI plays a stronger role in the outcome, while sex exerts no significant effect (adjusted *R*^2^ = 0.165). Multivariable regression analysis confirmed that BMI was a confounding factor and that sex itself was not an independent determinant of pre-WPUS PIS.

**Table 3:** Factors associated with the Pancreatic Image Scale on conventional ultrasonography

Variable	*β* (SE)	95% CI	*P*-value
Intercept	8.11 (0.71)	6.70–9.49	< 0.001
BMI	− 0.12 (0.02)	− 0.17 to − 0.08	< 0.001
Age	− 0.02 (0.01)	− 0.04 to − 0.02	< 0.001
Sex	0.30 (0.21)	− 0.10 to 0.71	0.14

Unstandardized regression coefficients (*β*) with 95% confidence intervals (CI) are presented. Factors included in the model were age, body mass index (BMI), and sex. For PIS on conventional ultrasonography (pre-WPUS), age (*β* =  − 0.02, *P* < 0.001) and BMI (*β* =  − 0.12, *P* < 0.001) were significant negative predictors, while sex showed no significant association (*β* =  0.30, *P* = 0.14; adjusted *R*^2^ = 0.165; *n* = 249)

Abbreviations: PIS, Pancreatic Image Scale; WPUS, water intake pancreatic ultrasonography; BMI, body mass index; *β*, regression coefficient; SE, standard error; CI, confidence interval

Regarding the effects of WPUS, sex was the only significant predictor of PIS improvement (Table 4), with women showing an average increase of 0.99 units compared with men (*P* < 0.001). Age (*P* = 0.82) and BMI (*P* = 0.22) were not significantly associated with PIS improvement. The model explained approximately 10% of the variance in PIS improvement (adjusted *R*^2^ = 0.09). Binary logistic regression (PIS improvement > 0 vs. ≤ 0) confirmed these findings: female sex remained the only significant predictor of PIS improvement (OR 5.45, 95% CI 2.22–13.30, *P* < 0.001), whereas BMI and age were not significantly associated (*P* = 0.61 and 0.33, respectively; Supplementary Table S1).

**Table 4:** Effect of WPUS on PIS improvement

Variable	*β* (SE)	95% CI	*P*-value
Intercept	2.44 (0.76)	0.95–3.94	0.001
BMI	− 0.03 (0.03)	− 0.09 to 0.02	0.22
Age	< 0.001 (0.01)	− 0.01 to 0.02	0.82
Sex	0.99 (0.22)	0.56–1.42	< 0.001

Multivariable linear regression analysis was performed to identify factors associated with improvement in the Pancreatic Image Scale (*n* = 249)

Regression coefficients (*β*) with 95% confidence intervals are presented

Abbreviations: WPUS, water intake pancreatic ultrasonography; PIS, Pancreatic Image Scale; BMI, body mass index; *β*, regression coefficient; SE, standard error; CI, confidence interval

We identified 14 pancreatic lesions during the study: one malignancy-suspected pancreatic lesion, one pancreatic neuroendocrine tumor, and 12 cystic lesions, including those suspected to represent intraductal papillary mucinous neoplasm (IPMN). WPUS newly detected six of the 12 cystic lesions (50%) that were not visible on baseline US. These cystic lesions corresponded to 1.7% of the consecutive 706 examinations.

Detailed characteristics and confirmation modalities are summarized in Table 2. A representative cystic lesion detectable only after WPUS is illustrated in Fig. 6. No patients experienced complications, such as abdominal pain or vomiting, during the water-drinking test.

**Fig. 6:**
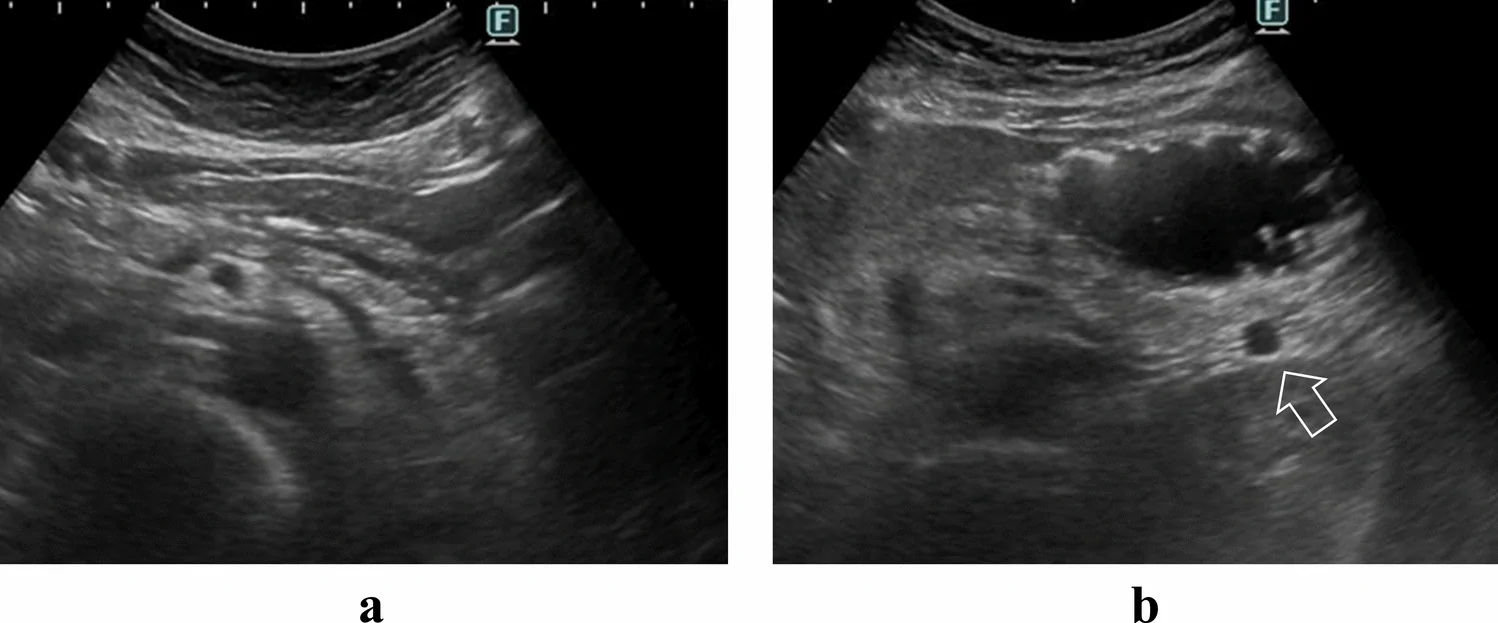
Cystic lesion visualized only after WPUS in a woman in her 50s. **a** Conventional transabdominal ultrasound (supine, pre-water; PIS = 4) failed to depict the pancreatic tail because of overlying gastric and intestinal gas, and no lesion was identified. **b** After water intake, WPUS in the supine position (WSP; PIS = 6) clearly demonstrated a 9-mm cystic lesion (arrow) in the pancreatic tail; similar visibility was obtained in the sitting position after water intake (WSit, not shown). Subsequent MRCP confirmed the lesion and revealed no concomitant lesion. Abbreviations: WPUS, water intake pancreatic ultrasonography; PIS, Pancreatic Image Scale; WSP, supine position after water intake; WSit, sitting position after water intake; MRCP, magnetic resonance cholangiopancreatography

### Study 2

In the reading of test cases by three gastroenterologists, ICCs for intraobserver reliability (1, 1) with reference to plain CT images were 0.85, 0.83, and 0.80 for each reader (95% CIs 0.50–0.86, 0.61–0.90, and 0.71–0.93, respectively). The interobserver correlation coefficient (ICC (3, 1)) was 0.71 (95% CI 0.55–0.84) at the initial reading and 0.61 (95% CI 0.41–0.77) 1 week later (Table 5). Regarding reproducibility, the scores assigned by the observers during the initial US readings correlated well with the corresponding CT scales. Although preliminary, these findings suggest that PIS provides a reasonably consistent measure for evaluating pancreatic visualization, with ICC values indicating acceptable intra- and inter-reader reliability.

**Table 5:** Study 2—Intra- and interobserver reliability of the Pancreatic Image Scale: intraclass correlation coefficients under three reading conditions

Reading conditions	Reader A ICC (1, 1)	Reader B ICC (1, 1)	Reader C ICC (1, 1)	Interobserver agreement ICC (3, 1)
Initial reading	–	–	–	0.71
Re-reading CT referenced	0.85	0.83	0.80	0.71
Re-reading After 1 week	0.73	0.80	0.85	0.61

Three reading conditions were evaluated: the initial reading, a CT-referenced second reading, and a third reading performed one week later. Intra-reader reliability for each reader (A–C) is presented as ICC (1,1), and interobserver agreement among the three readers is presented as ICC (3,1)

Abbreviations: PIS, Pancreatic Image Scale; ICC, intraclass correlation coefficient; CT, computed tomography

## Discussion

### Effect of WPUS

This study yielded two key findings. First, WPUS significantly expanded the visualizable range of the pancreas, with greater improvements observed in female patients. Second, Study 2 confirmed the reproducibility of the PIS, supporting its utility as a quantitative tool to evaluate pancreatic visibility. Although previous studies reported better pancreatic visualization in women using conventional US [2, 17], our findings indicate that this apparent advantage reflects the lower BMI distribution among women in our cohort rather than an independent effect of sex. These discrepancies are likely attributable to differences in study populations and approaches to controlling confounding factors. In our study, BMI was identified as the primary factor influencing pre-WPUS visualization, whereas sex was not an independent determinant. This BMI-related sex difference is also reflected in nationwide data from the Ministry of Health, Labour, and Welfare of Japan (Supplementary Fig. S4) [18], suggesting population-level rather than cohort-specific bias.

WPUS has been proposed as a simple method to enhance pancreatic visualization. Several studies were limited by small sample sizes or relied on qualitative assessments [6, 11]. Others applied WPUS in high-risk groups and reported favorable outcomes [7–10]; however, the direct quantitative effect in unselected outpatients has not been fully established. As a fundamental step, this study quantified the degree of visualization improvement and identified patient- and technique-related determinants. The differences in study population, outcome measures, and evaluation methods indicate that our results complement rather than contradict the existing literature. We also observed greater improvement in the visualization of the pancreatic body and tail among women. To our knowledge, sex-specific effects have not been systematically evaluated in previous WPUS studies, and this hypothesis-generating observation warrants confirmation in prospective studies. This sex-related pattern was not apparent in routine practice despite years of WPUS use and became evident only through statistical analysis of a large cohort. However, our dataset lacked laboratory profiles and objective assessments of gastric configurations. A speculative but plausible explanation is that female-predominant anatomical features, such as gastric ptosis, may shift or enlarge the acoustic window and enhance the benefit of water intake for US imaging [19].

### Mechanistic interpretation

WPUS improves visualization when the fluid-filled stomach overlies the pancreas, because water intake creates an acoustic window, enhances acoustic coupling, and reduces attenuation and scattering. The efficacy of WPUS, therefore, depends partly on the relative anatomical positioning of the stomach and pancreas. When the stomach is anatomically adjacent to the pancreatic body and tail (e.g., large or caudally positioned stomach), visualization improves markedly, whereas when gas-filled bowel loops or a distant gastric remnant are interposed, the benefit may be smaller. This anatomical dependence may also explain the variability in WPUS improvement observed among patients. This mechanism may partly explain why WPUS showed greater improvement in women in Study 1 as women tend to have a more caudally positioned or more mobile stomach (e.g., gastric ptosis), increasing the likelihood that a fluid-filled stomach lies anterior to the pancreas or moves closer to it during positional change, thereby functioning as an acoustic window.

### Clinical utility of the PIS

Study 2 demonstrated at least moderate intra- and interobserver reproducibility of the PIS, providing preliminary validation of the scale as a practical tool for quality assessment in pancreatic US. By offering an objective and standardized metric, PIS may help reduce operator dependence and inform clinical decision-making; for example, distinguishing adequately visualized examinations from those requiring further evaluation with CT or MRI. Comparable to the Boston Bowel Preparation Scale used in colonoscopy [20, 21], the PIS provides a quantitative framework for assessing image quality. Our findings align with those of previous reports showing reduced pancreatic tail visibility at higher BMIs [2, 17]. The apparent sex-related differences disappeared after adjustment for BMI, reinforcing the validity of PIS-based interpretation. Taken together, this reading experiment was exploratory and supplementary, and was not designed for a detailed comparison of diagnostic performance among readers.

### Pancreatic tail visualization

This study focused on the pancreatic body and tail. Although cancers in these regions are less frequent, population-based registries indicate that tail-confined cancers represent approximately 15–20% of cases, while lesions involving the body and tail constitute 25–35% of all pancreatic cancers [22–24]. Reports suggest that tumors in the tail may exhibit more aggressive biology [25], and it is clinically relevant that malignant lesions in the tail, being anatomically farther from major central vascular structures than those in the head or body, may be more amenable to surgical treatment. Therefore, these regions should not be overlooked in routine evaluation. Figure 6 illustrates a representative case: a tail lesion not visible on conventional US became detectable after water intake. When relying solely on conventional US without WPUS, lesions in this region are at risk of being missed.

### Incremental diagnostic yield provided by WPUS

Pancreatic cancer is rarely detected on abdominal US in population-based screening, and the identification of a single malignancy-suspected lesion in our cohort is consistent with nationwide detection rates (0.005%) [9]. In addition, six cystic lesions (0.8%) were detected on baseline US alone, whereas the addition of WPUS increased the total number of detected cystic lesions to 12 (1.7% of all consecutive 706 examinations). Approximately half of these lesions were detected only after the addition of WPUS, underscoring its potential to reveal findings that may be overlooked by standard US. Taken together, these findings suggest that, in this cohort, the addition of WPUS may approximately double the number of detectable pancreatic cystic lesions compared with standard US alone. This detection rate falls within the low single-digit range reported in a large general-population screening study using abdominal US (1.3% among 9369 examinees) [26], although differences in study populations and confirmation protocols preclude direct comparison of diagnostic sensitivity. Therefore, these figures should be interpreted as reference values rather than comprehensive estimates. One pancreatic head lesion was also newly identified with WPUS, suggesting that its diagnostic contribution may extend beyond the body and tail.

Although WPUS adds approximately 6–9 min to the examination, including water ingestion, this additional time is considered justified by its diagnostic benefit, and it remains a well-tolerated, noninvasive, and cost-effective technique with high feasibility that can be readily implemented even in general outpatient practices without specialized equipment or advanced expertise. With modest workflow refinements—including streamlining body-position protocols—WPUS could be applied more efficiently and may be suitable for wider use, even in screening contexts where examination time is critical.

In summary, WPUS represents a valuable adjunct to conventional pancreatic US and may enable more comprehensive evaluation of the entire pancreas. Broader adoption of WPUS and refinement of the PIS as a standardized quality metric may further enhance its clinical utility. Beyond conventional early indicators of pancreatic cancer, such as pancreatic cysts and main pancreatic duct dilatation [7–10], fatty pancreas (pancreatic fat infiltration) is emerging as a potentially relevant US finding recently [27–29]. WPUS may facilitate the recognition of such risk factors and support risk stratification and follow-up in clinical practice, warranting further prospective evaluations.

## Limitations

This study had several limitations. First, it was a retrospective, single-center study, and both US acquisition and PIS assignment in Study 1 were performed by a single experienced operator. This ensured consistency in image acquisition and scoring but precluded assessment of inter-operator variability in WPUS performance. In addition, because the study spanned a long real-world clinical period, progressive learning and refinement of scanning techniques over time may have influenced image interpretation.

Second, the data were obtained during routine clinical practice; therefore, unintentional case selection and publication bias cannot be excluded. Moreover, the sonographer was not blinded to clinical information as complete operator blinding was not feasible in daily practice. BMI was not available in all cases, introducing potential selection bias in analyses involving BMI. Only three patients had prior gastrectomy, so the impact of altered anatomy on WPUS performance could not be assessed. In addition, WPUS was not performed in symptomatic patients or in those who had undergone total gastrectomy as they were considered unsuitable for water intake.

Third, with respect to Study 2, the images used were real-world clinical images, which increases clinical applicability but entails variability in image quality and a potential case-selection bias. In particular, the reading experiment used 30 cases selected from examinations with stored CT images, which limits representativeness. Moreover, the sample size for the reproducibility analysis was modest.

Finally, the PIS is an ordinal scale, yet it was treated as a continuous variable in some regression analyses, which may impose model-related constraints. Future work should include multicenter, prospective validation involving multiple operators and standardized acquisition and scoring protocols to confirm the external applicability and diagnostic utility of WPUS and the PIS.

## Conclusion

This quantitative study demonstrated that combining water intake with positional adjustments, including the right-lateral decubitus position (WPUS), significantly expanded the US visualization range of the pancreatic body and tail compared with conventional methods. Implementing WPUS in clinical practice may potentially increase the diagnostic sensitivity of conventional US. In addition, the newly introduced PIS enabled objective and reproducible assessment and proved useful as a quality control indicator in routine clinical practice. Furthermore, quantitative analysis revealed no sex differences in baseline visualization but showed greater improvements with WPUS in women. Although further validation is warranted, these findings suggest that WPUS and PIS may contribute to more consistent and comprehensive pancreatic imaging, even in the general outpatient population.

## Supplementary Information

Below is the link to the electronic supplementary material.

Supplementary file1 (TIF 1704 KB)

Supplementary file2 (TIF 1752 KB)

Supplementary file3 (TIF 1434 KB)

Supplementary file4 (TIF 1449 KB)

Supplementary file5 (DOCX 21 KB)

## Abbreviations

AUS: Abdominal ultrasonography
US: Ultrasonography/ultrasound
BMI: Body mass index
CECT: Contrast-enhanced computed tomography
CI: Confidence interval
ICC: Intraclass correlation coefficient
MRCP: Magnetic resonance cholangiopancreatography
OR: Odds ratio
PIS: Pancreatic Image Scale
PV: Portal vein
SMV: Superior mesenteric vein
SV: Splenic vein
WPUS: Water intake pancreatic ultrasonography
IPMN: Intraductal papillary mucinous neoplasm
