# Supplementary material for: Water intake pancreatic ultrasonography improves pancreatic body and tail visualization: contributing factors revealed by quantitative analysis
Source: J Med Ultrason (2001). 2026 May 9;53(4):439–49. doi: 10.1007/s10396-026-01622-y (PMC13694480; doi:10.1007/s10396-026-01622-y)
Supplement: Supplementary file 1 [file 10396_2026_1622_MOESM5_ESM.docx]

**Figure S1. Patient-selection flow.**

A total of 706 consecutive water intake pancreatic ultrasonography examinations were screened, and 581 patients with complete Pancreatic Image Scale scores were included in the main dataset. From these, three analytic subsets were derived: positional analysis (n = 263, seven-position data*), PIS improvement versus body mass index analysis (n = 90, seven-position + BMI), and multivariable predictor analysis (n = 249, BMI available).

*Seven-position protocol cases only.

Abbreviations: WPUS, water intake pancreatic ultrasonography; PIS, Pancreatic Image Scale; BMI, body mass index.

**Figure S2. Distribution of PIS in the SP position (pre-WPUS) by sex.**

Histogram showing Pancreatic Image Scale values before water intake in the SP (supine) position. Female participants had higher PIS values than male participants, suggesting sex-related differences in depictability. This apparent difference prompted further analyses to examine possible confounding factors.

Abbreviations: PIS, Pancreatic Image Scale; SP, supine position; WPUS, water intake pancreatic ultrasonography.

**Figure S3. Distribution of BMI in this study cohort by sex.**

Histogram of body mass index values for male and female participants. Men tended to have a higher BMI than women, indicating that the apparent sex difference in Pancreatic Image Scale values (Figure S2) may be partly attributable to differences in body habitus.

Abbreviations: BMI, body mass index.

**Figure S4. Distribution of BMI in the general Japanese population by sex.**

Histogram of body mass index in Japanese men and women based on the National Health and Nutrition Survey, 2022 (Ministry of Health, Labour, and Welfare, Japan; available from https://www.mhlw.go.jp/toukei/itiran/gaiyo/k-eisei.html) and the government statistics portal e-Stat (https://www.e-stat.go.jp/). The national dataset also showed sex differences in the BMI distribution, supporting the interpretation that body habitus contributes to the observed sex-related differences in Pancreatic Image Scale values.

Abbreviations: BMI, body mass index.

**Table S1. Logistic regression analysis of PIS improvement.**

A binary logistic regression model (ΔPIS > 0 vs. ≤ 0) was used to evaluate predictors of PIS improvement. Female sex remained the only significant predictor (OR = 5.45, 95% CI: 2.22–13.30, P < 0.001), whereas BMI and age were not associated with PIS improvement (P = 0.61 and 0.33, respectively; n = 249).

| Variable | OR | 95% CI | P-value |
| --- | --- | --- | --- |
| BMI | 0.98 | 0.89–1.07 | 0.61 |
| Age | 0.99 | 0.96–1.01 | 0.33 |
| Sex | 5.45 | 2.22–13.3 | **< 0.001** |

Abbreviations: PIS, Pancreatic Image Scale; OR, odds ratio; CI, confidence interval; BMI, body mass index.
